# Impact of behavioral insights-informed social media campaigns on HPV vaccination in Bangladesh

**DOI:** 10.1371/journal.pgph.0006732

**Published:** 2026-06-23

**Authors:** Sohail Agha

**Affiliations:** 1 Department of Epidemiology, University of Washington, Seattle, Washington, United States of America; 2 Behavioral Insights Lab, Seattle, Washington, United States of America; University of Michigan, UNITED STATES OF AMERICA

## Abstract

Cervical cancer remains a leading cause of cancer-related deaths among women in Bangladesh. In 2024, the government introduced the single-dose HPV vaccine across six divisions of Bangladesh through a school-based vaccination campaign. This study evaluates the impact of targeted and population-wide social media interventions designed to boost HPV vaccine uptake. A pre-test/post-test quasi-experimental study was conducted across six divisions of Bangladesh with four intervention groups and one control group. Caregivers of adolescent girls aged 9–17 were recruited for baseline (n = 3,823) and follow-up (n = 7,004) online surveys. Interventions included: (T1) targeted social media plus school awareness, (T2) targeted social media only, (T3) school awareness only, and (T4) population-wide social media. Logistic regression models with interaction terms were used to assess intervention effects. HPV vaccination coverage increased from 10.1% to 32.9% in the control group, consistent with the national campaign being implemented across all study areas. Adjusted odds ratios showed significant additional effects for T1 (OR = 1.52, 95% CI: 1.30–1.77), T2 (OR = 1.92, 95% CI: 1.40–2.65), T3 (OR = 1.08, 95% CI: 1.01–1.16), and T4 (OR = 1.36, 95% CI: 1.28–1.45). Adjusted marginal effects indicated that, relative to the control group, the probability of vaccination increased by 11.8 percentage points for the targeted social media intervention and 5.3 percentage points for the population-wide social media intervention. The school-awareness intervention was associated with a 1.3 percentage point increase. Each behavioral insights-informed social media intervention was associated with a measurable impact on HPV vaccination beyond that of the national campaign. The targeted social media intervention was associated with twice as large an increase in coverage as the population-wide social media intervention (11.8 versus 5.3 percentage points). Insights-informed social media interventions can play an important role in national vaccine introductions in low- and middle-income countries.

## Introduction

Human papillomavirus (HPV) is a significant cause of cervical cancer, which remains one of the leading causes of cancer-related deaths among women in Bangladesh [1]. Population-level analyses demonstrate that HPV vaccine interventions that achieve high coverage can substantially reduce the burden of cervical cancer and save lives [2].

Mass vaccination campaigns have a long history in LMICs, including Bangladesh, where they have been instrumental in addressing vaccine-preventable diseases. For example, the 2014 Measles-Rubella (MR) campaign in Bangladesh achieved over 90% coverage, [3] while similar efforts in India and Nepal reported vaccination rates of 80% and 85%, respectively [4,5].

Despite these successes, more recent mass vaccination campaigns have faced significant hurdles, exacerbated by misinformation and vaccine hesitancy stemming from the COVID-19 pandemic. For example, COVID-19 vaccination campaigns in Nigeria reported only 54% coverage after several months of a mass vaccination effort, [6] and ongoing polio campaigns in Pakistan and Afghanistan have achieved only 40–60% coverage in some districts [7,8]. In Afghanistan, estimated coverage of three doses of oral poliovirus vaccine (OPV) among children aged 1 year was 56% in 2021, while coverage of two doses of inactivated poliovirus vaccine (IPV) was 45% in 2023.

HPV vaccination campaigns have experienced uneven success in sub-Saharan Africa. A 2019–2020 national HPV vaccine campaign in Kenya using a two-dose regimen reached only 33% coverage for the second dose [9]. By contrast, almost a decade earlier, Rwanda demonstrated exceptional success in implementing its national HPV vaccination program. Owing to strong political commitment and comprehensive community, and school-based vaccine delivery, Rwanda achieved over 93% vaccine coverage with the three-dose HPV vaccine regimen in 2011 [10]. Lessons from Rwanda highlight the importance of sustained political will and community engagement, critical for addressing challenges in South Asia, where HPV vaccination rates have remained below 10%.

Few published studies in South Asia have used surveys to estimate HPV vaccine prevalence following mass vaccination campaigns. One study that estimated HPV vaccine prevalence following school-based mass vaccination campaign in Dhaka Division in 2023, found that 33% of adolescent girls had received the HPV vaccine [11]. Pre-campaign estimates suggested low prevalence of HPV vaccination in Bangladesh [12]: a systematic review and meta-analysis reported pooled HPV vaccination prevalence at approximately 8% in the region, with Bangladesh (3%), India (6%), and Pakistan (4%) [12] falling below the regional average. Understanding the extent to which HPV vaccination efforts in South Asia are successful is important, particularly given the recent challenges faced by HPV vaccine introduction in other countries in the region, as reported in national media [13].

Common challenges faced by mass HPV vaccination campaigns in LMICs have included difficulty in reaching remote areas and maintaining cold-chain infrastructure. Vaccine hesitancy, driven by misinformation about side effects (including infertility concerns) and low perceived disease risk further hinder uptake. Operational challenges, such as overestimating vaccine coverage based on vaccine distribution numbers rather than population-level estimates, also compound these issues [12]. For instance, independent monitoring in Zambia revealed significant barriers to vaccine delivery, including parental concerns and logistical inefficiencies. Findings from both sub-Saharan Africa and South Asia underscore the need to integrate robust monitoring mechanisms in mass vaccination campaigns [14].

### HPV vaccination in Bangladesh: From demonstration project to national scale-up

Prior to national introduction, Bangladesh implemented a demonstration HPV vaccination project in Gazipur District in 2016 to assess the feasibility of including the HPV vaccine in the national immunization program [15]. The intervention targeted a limited population of approximately 30,000 grade 5 schoolgirls and was implemented over several months, reflecting a routine, high-intensity delivery model rather than a short campaign. Adolescent girls received two doses of the HPV vaccine administered six months apart, delivered through a combination of school-based sessions, routine EPI fixed sites, and community outreach. A WHO post-introduction evaluation found that the Gazipur pilot achieved 94% vaccination coverage, demonstrating strong acceptability and effective service delivery under controlled conditions [15].

In contrast, the subsequent national introduction of HPV vaccination across Bangladesh in 2023–2024 [11] involved a vastly larger and more heterogeneous target population and relied on short-duration, campaign-style delivery. To enhance reach and impact at scale, the national rollout was accompanied by multimedia and social media–based communication efforts, which sought to increase caregiver awareness, motivation, and timely uptake within a compressed implementation window. The Gazipur experience thus represents a small-scale, intensive, and prolonged pilot, whereas the national introduction reflects the challenges and opportunities of rapid scale-up, where digital and social media strategies are leveraged to augment traditional public health delivery systems.

Beginning with an 18-day Multi-Age Cohort (MAC) campaign in Dhaka Division, the Government of Bangladesh initiated a phased introduction of the WHO-recommended single dose HPV vaccine in 2023. A post-test evaluation survey found that one-third of adolescent girls in Dhaka Division had received the HPV vaccine, suggesting that significant barriers to uptake remained after the mass vaccination effort, including logistical challenges and uneven exposure to the campaign [11].

In 2024, political disturbances delayed the phased rollout of the HPV vaccine in the remaining six divisions of Bangladesh. The national vaccination campaign was implemented in the remaining divisions once the new government was formed. Four interventions were implemented in coordination with the HPV vaccine introduction to increase the impact of the national campaign: (1) a targeted social media campaign combined with an in-school awareness campaign, (2) a targeted social media campaign alone, (3) a school awareness campaign alone, and (4) a population-wide social media campaign. These four interventions are referred to throughout this paper as treatment arms: Treatment 1 (T1), Treatment 2 (T2), Treatment 3 (T3), and Treatment 4 (T4), where each label denotes a distinct intervention type. In the first three divisions—Barisal, Chittagong, and Sylhet—the targeted social media intervention delivered advertisements to caregivers of adolescent girls located within a one-kilometer radius of participating schools. Within these divisions, the targeted social media campaign was combined with an in-school awareness intervention in Treatment 1 (T1), implemented alone in Treatment 2 (T2), and the in-school awareness campaign alone was implemented in Treatment 3 (T3).

In the three remaining divisions, Khulna, Rajshahi, and Rangpur, a population-wide social media intervention was implemented. Both social media campaigns employed identical content. Posts and videos were based on behavioral insights derived from a survey conducted across all six divisions in 2023. The primary difference between the social media interventions implemented in the first three divisions and the remaining three divisions was that the population-wide social media intervention was not restricted to caregivers who lived within 1 Km of schools.

#### Study objective.

This study uses a quasi-experimental pre-test/post-test design to assess the additional impact of social media interventions on HPV vaccine uptake during a national vaccination campaign that introduced the single-dose HPV vaccine in six divisions of Bangladesh. The study also examines the effect of an in-school awareness campaign implemented to support the vaccine introduction.

## Methods

### Ethics statement

This study received ethical approval from the Institutional Review Board of the Institute for Developing Science and Health Initiatives (ideSHi), Bangladesh (Protocol #PNR-23003). The original approval was granted on 29 August 2023, and an addendum to the protocol was approved on 28 October 2023. These approvals covered the full study period, including both baseline and follow-up data collection.

All participants were caregivers aged 18 years or older. Participation was voluntary, and informed consent was obtained electronically prior to survey initiation. The study collected information about adolescent girls aged 9–17 from their adult caregivers; no data were collected directly from children or from individuals under 18 years of age.

All procedures adhered to the Declaration of Helsinki, and all data were anonymized prior to analysis.

### Study design

Led by the Bangladesh Ministry of Health, the national campaign to promote HPV vaccination consisted of a mass media and community outreach initiative in the six divisions of Bangladesh mentioned earlier (Barisal, Chittagong, Sylhet, Khulna, Rajshahi, Rangpur). This study evaluates the impact of the social media and in-school awareness interventions on vaccine uptake during the national level introduction of the single dose HPV vaccine.

Per the 2022 Census of Bangladesh, these six divisions comprise two thirds of the population of Bangladesh, or 109 million out of 165 million people. As mentioned earlier, the national campaign was initiated in Dhaka Division in 2023 [11], and the phased introduction of the vaccine in the remaining divisions was delayed until 2024. The study utilized a pre-test/post-test quasi-experimental design to evaluate the impact of social media and school-based interventions on HPV vaccination uptake.

Public schools in Barisal, Chittagong, and Sylhet divisions were randomly assigned to either Treatment 1, Treatment 2, Treatment 3, or the control group. Treatment 1 consisted of a social media campaign delivered to caregivers located within a 1 km area around intervention schools, combined with an in-school campaign to increase adolescent girls’ awareness of HPV vaccination. Treatment 2 comprised a social media campaign alone, delivered to caregivers located within a 1 km area around intervention schools. Treatment 3 consisted of an in-school campaign alone to increase awareness of HPV vaccination among adolescent girls. The control group comprised schools in Barisal, Chittagong, and Sylhet divisions where neither the social media nor the school awareness intervention was implemented; the control group was exposed only to the national vaccine rollout activities.

In Khulna, Rajshahi, and Rangpur divisions, a population-wide social media intervention was implemented. The population-wide social media campaign reached users across the three divisions. It did not target caregivers within 1 km area around schools. This comprised Treatment 4. The behavioral insights-informed social media content was identical across all treatment groups that included a social media component (Treatment 1, Treatment 2, and Treatment 4). Table 1 provides a description of each intervention.

**Table 1:** Description of Control and Intervention Groups.

Group	Description of Intervention	Division(s)	Primary Delivery Mechanism
**Control (C)**	National HPV vaccination campaign only; no additional social media or school awareness activities.	Barisal, Chittagong, Sylhet	Standard government school-based mass vaccination program.
**Treatment 1 (T1)**	Targeted social media intervention delivered within a 1 km radius of intervention schools, combined with an in-school awareness campaign to increase adolescent girls’ awareness of HPV vaccination.	Barisal, Chittagong, Sylhet	Facebook/Instagram posts and videos + in-school awareness activities.
**Treatment 2 (T2)**	Targeted social media intervention delivered within a 1 km radius of intervention schools (no in-school awareness activities).	Barisal, Chittagong, Sylhet	Facebook/Instagram posts and videos.
**Treatment 3 (T3)**	In-school awareness campaign to increase awareness of HPV vaccination among adolescent girls (no social media intervention).	Sylhet	In-school awareness activities
**Treatment 4 (T4)**	Population-wide social media intervention reaching caregivers across entire divisions (no geographic targeting).	Khulna, Rajshahi, Rangpur	Facebook/Instagram posts and videos.

The quasi-experimental design therefore consisted of four intervention groups and one control group implemented across six divisions, allowing comparison of targeted social media, population-wide social media, and school-based awareness interventions relative to areas exposed only to the national campaign.

Although schools in Barisal, Chittagong, and Sylhet divisions were randomly assigned to treatment conditions, the study is characterized as quasi-experimental rather than a cluster-randomized trial for several reasons. First, caregivers were not enrolled through the school clusters — they were recruited independently via Meta advertisements targeted geographically to the catchment areas of participating schools. Caregiver participation was determined by self-selection, meaning that the unit of randomization (school) and the unit of recruitment and analysis (individual caregiver) are distinct. Second, the design is repeated cross-sectional: baseline and follow-up respondents are not the same individuals, unlike a true cluster-randomized trial in which the same participants are followed over time. Third, Treatment 4 was allocated at the division level rather than the school level, representing a different and non-randomized allocation mechanism from T1–T3; the three divisions receiving T4 were geographically distinct from the three divisions containing T1–T3 and the control group, introducing potential contextual differences that school-level randomization did not control for. To account for the discrepancy between the unit of assignment (school) and the unit of analysis (individual caregiver), cluster-robust standard errors were estimated, clustering observations at the division level.

### Behavioral insights to design the social media intervention

A foundational behavioral insights survey was conducted in November-December 2023 with 2,369 caregivers of adolescent girls across the six divisions of Bangladesh included in this study. Designed using the Fogg Behavior Model, [16] the survey gathered data on caregivers’ motivation, ability, and social norms related to HPV vaccination. The primary outcome variable was caregivers’ expectation that their child would receive the HPV vaccination in the next 12 months. These findings directly informed the design of the social media posts and videos.

Findings from the behavioral insights study revealed that:

- Caregivers who believed the vaccine promised their daughters a brighter future were 8 percentage points more likely to expect that their child would get vaccinated within the next 12 months.
- Perceived social norms were strongly associated with vaccination expectations. There was a 6 percentage-point difference in expectations that the child would get vaccinated between caregivers who perceived family and community approval of HPV vaccination versus those who did not.
- Caregivers whose daughters expressed interest in getting vaccinated showed a 21 percentage-point higher expectation that their child would get vaccinated.

### Interventions

1. Social Media Campaign

Caregivers in Treatment 1, Treatment 2 and Treatment 4 were reached through advertisements on Facebook and Instagram. The ads featured a link to the Bangladesh Ministry of Health’s HPV vaccine registration site (http://vaxepi.gov.bd/). Registration on this site was required for a girl to receive the HPV vaccine. The social media campaign focused on three key themes that emerged from the foundational behavioral insights research conducted in 2023. These insights were used to design ads to increase adoption of the HPV vaccine.

Digital campaign metrics such as impressions and exposure frequency are commonly reported in digital media campaigns because they indicate how often advertisements are displayed on users’ screens. However, impressions represent opportunities for exposure rather than confirmed engagement with the message and cannot be directly linked to individual behavioral outcomes. Because the objective of this study was to evaluate whether targeted digital messaging influenced vaccination behavior, the analysis focuses on individual-level vaccination outcomes rather than platform-level exposure metrics.

The social media campaign focused on three key themes:

- HPV vaccination enables a brighter future by protecting girls’ health.
- Communities approve of vaccination.
- Girls are active participants in their health decisions.

2. School awareness Program

In-school awareness raising activities in Treatment 3 (and Treatment 1) included the following:

- Interactive discussions with girls on health decisions.
- Quizzes and information sessions on HPV vaccine.
- Pre- and post-event surveys to assess changes in knowledge and attitudes.
- Counseling sessions to address caregivers’ concerns and to provide logistical details.

### Measures

- Outcome: Vaccination status of the adolescent girl, reported by their caregiver.
- Independent Variables: Socio-demographic characteristics, including caregiver age, education, gender, residence (urban, rural), and the adolescent girl’s age.

### Study population and sampling strategy

The study population consisted of caregivers of girls aged 9–17 from both urban and rural areas across the six divisions.

A stratified random sampling approach was employed, stratified by division, urban/rural residence and gender. Participants were recruited through Meta social media advertisements which targeted caregivers based on their online activity, location, and demographic profile. The follow-up sample sizes were adjusted due to recruitment challenges at baseline in Treatment 2 and the control group.

Table 2 provides the estimated audience, obtained from Meta’s audience insights tool. The control area had an estimated audience of 5.9 million people on Facebook and Instagram. Treatment 1 had an estimated audience of about 6.2 million people. Treatment 2 was smaller than control or Treatment 1, with an estimated audience of 1.6 million. Treatment 3 was the smallest with an estimated audience of about 0.7 million. Treatment 4 was the largest with an estimated audience of 12.3 million.

**Table 2:** Estimated Audience and Sample Sizes of Control and Treatment Groups.

	Description of Intervention	Estimated Audience (Meta)	Baseline Survey		Follow-up Survey	
			Planned (n)	Actual (n)	Planned (n)	Actual (n)
**Control (C)**	National HPV vaccination campaign only	5,881,100	500	159	1,000	1,003
**Treatment 1 (T1)**	Targeted social media intervention (within 1 km of schools) + in-school awareness campaign	6,214,600	500	786	1,000	1,074
**Treatment 2 (T2)**	Targeted social media intervention only (within 1 km of schools)	1,644,100	500	754	1,000	1,190
**Treatment 3 (T3)**	School awareness campaign only (no social media component)	671,800	500	44	500	516
**Treatment 4 (T4)**	Population-wide social media intervention (division-level targeting)	12,300,000	2,000	2,080	3,000	3,221

For the baseline survey, we aimed to collect data from 4,000 respondents: 500 each in the control group, Treatment 1, Treatment 2, and Treatment 3, and 2,000 in Treatment 4. Due to the formation of a new government in Bangladesh in 2024 and the unexpected delay in the introduction of the HPV vaccine in these six divisions, there was an urgent need to conduct the national campaign as early as possible. This meant that the time available to conduct the baseline survey before the start of the national campaign was limited to two weeks.

Given the time constraint, a sample size of 500 respondents each for treatment and control groups seemed feasible for the baseline survey. For the follow-up survey, we planned for larger sample sizes to compensate for smaller sample sizes at baseline: 1,000 respondents each in the control group, Treatment 1, and Treatment 2; 500 in Treatment 3; and 3,000 in Treatment 4, for a total planned follow-up sample of 6,500 caregivers.Assuming a baseline HPV prevalence of 5%, the planned baseline (n = 500) and follow-up (n = 1,000) sample sizes provided approximately 80% power to detect a 3.6 percentage-point change in HPV vaccination between baseline and follow-up for the Control, Treatment 1, and Treatment 2 groups (two-sided α = 0.05).

For Treatment 3, which had a smaller planned follow-up sample size (n = 500), the study had 80% power to detect a 5.1 percentage-point change in HPV vaccination between baseline and follow-up. For Treatment 4, with larger planned sample sizes at baseline (n = 2,000) and follow-up (n = 3,000), the minimum detectable change was correspondingly smaller—approximately 1.8 percentage points at 80% power (two-sided α = 0.05).

These calculations assume independent samples at baseline and follow-up and were designed to ensure sufficient statistical power to detect programmatically meaningful changes in HPV vaccination coverage across all study arms.

### Data collection

The baseline survey was conducted from 23/09/2024 to 8/10/2024. The unpredictability in how the Meta ad algorithm functions affected the baseline sample sizes in the control group and Treatment 3. The Meta algorithm approves and releases ad sets in stages. The ad sets for Treatment 1 and Treatment 2 were released on September 23, 2024, allowing 15 days for data collection before October 8, when the survey had to be stopped due to preparations for the national campaign. However, the ad sets for the control group and Treatment 3 were not released until October 1, 2024, leaving only seven days for baseline data collection. Interviews were completed with 159 caregivers in the control group and 44 respondents in Treatment 3 during the seven days available for the baseline data collection for these two groups.

Although baseline sample sizes differed across study groups due to the staggered release of advertisement sets by Meta’s algorithm, the analysis pooled baseline and follow-up observations and used multivariate logistic regression models adjusting for socio-demographic characteristics and clustering within intervention areas. This approach reduces the risk that differences in baseline sample sizes bias the estimated intervention effects. Despite the smaller baseline samples in the control group and Treatment 3, the distributions of key caregiver and adolescent characteristics were broadly similar across study groups (Table 3), suggesting that the baseline samples remained reasonably comparable.

**Table 3:** HPV Vaccination Levels and Caregiver Characteristics from Baseline and Follow-up Surveys.

	C		T1		T2		T3		T4	
	Baseline	Follow-up	Baseline	Follow-up	Baseline	Follow-up	Baseline	Follow-up	Baseline	Follow-up
**Adolescent vaccinated**										
No	143 (89.9)	673 (67.1)	735 (93.5)	722 (67.2)	709 (94.0)	769 (64.6)	39 (88.6)	323 (62.6)	1904 (91.5)	2056 (63.8)
Yes	16 (10.1)	330 (32.9)	51 (6.5)	352 (32.8)	45 (6.0)	421 (35.4)	5 (11.4)	193 (37.4)	176 (8.5)	1165 (36.2)
**Age of caregiver**										
18-29	35 (22.0)	393 (39.2)	141 (17.9)	407 (37.9)	163 (21.6)	485(40.8)	14 (31.8)	209 (40.5)	701 (33.7)	1253 (38.9)
30-39	83 (52.2)	353 (35.2)	403 (51.3)	359 (33.4)	379 (50.3)	382 (32.1)	18 (40.9)	177 (34.3)	811 (39.0)	1098 (34.1)
40 and older	41 (25.8)	257 (25.6)	242 (30.8)	308 (28.7)	212 (28.1)	323 (27.1)	12 (27.3)	130 (25.2)	568 (27.3)	870 (27.0)
**Gender of caregiver**										
Male	87 (54.7)	534 (53.2)	514 (55.4)	580 (54.0)	429(56.9)	600 (50.4)	29 (65.9)	270 (52.3)	1062 (51.1)	1677 (52.1)
Female	72 (45.3)	469 (46.8)	272 (34.6)	494 (46.0)	325 (43.1)	590 (49.6)	15 (34.1)	246 (47.7)	1018 (48.9)	1544 (47.9)
**Education of caregiver**										
Less than higher secondary	48 (30.2)	346 (34.5)	274(34.9)	382 (35.6)	252 (33.4)	431 (36.2)	13 (29.5)	180 (34.9)	569 (27.4)	990 (30.7)
Higher secondary	36 (22.6)	292 (29.1)	205 (26.1)	268 (25.0)	177 (23.5)	296 (24.9)	11 (25.0)	134 (26.0)	522 (25.1)	866(26.9)
Bachelor’s	32 (20.1)	189 (18.8)	128 (16.3)	207 (19.3)	121 (16.0)	240 (20.2)	8 (18.2)	107 (20.7)	425 (20.4)	669 (20.8)
Master’s	43 (27.0)	176 (17.6)	179 (22.8)	217 (20.2)	204 (27.1)	223 (18.7)	12 (27.3)	95 (18.4)	564 (27.1)	696 (21.6)
**Age of adolescent**										
9-11	95 (59.8)	529 (52.7)	548 (69.7)	576 (53.6)	1061 (68.9)	1237 (54.6)	20 (45.5)	279 (54.1)	1329 (63.9)	1757 (54.6)
12-14	43 (27.0)	269 (26.8)	168 (21.4)	301 (28.0)	332 (21.6)	616 (27.2)	14 (31.8)	152 (29.5)	487 (23.4)	892 (27.7)
15-17	21 (13.2)	205 (20.4)	70 (8.9)	197 (18.3)	147 (9.6)	411 (18.2)	10 (22.7)	85 (16.5)	264 (12.7)	572 (17.8)
**Residence**										
Urban	102(64.2)	565 (56.3)	430 (54.7)	673 (62.7)	365 (48.4)	729 (61.3)	27 (61.4)	336 (65.1)	1094 (52.6)	1671 (51.9)
Rural	57 (35.8)	438 (43.7)	356 (45.3)	401 (37.3)	389 (51.6)	461(38.7)	17 (38.6)	180 (34.9)	986 (47.4)	1550 (48.1)
**Total**	159 (100.0)	1003 (100.0)	786 (100.0)	1074 (100.0)	754 (100.0)	1190 (100.0)	44 (100.0)	516 (100.0)	2080 (100.0)	3221 (100.0)

Values are n (%).

Since the algorithm approved ads for the baseline survey in Treatment 1 first, a larger number of caregivers were interviewed in Treatment 1 and Treatment 2 than originally planned: 786 caregivers were interviewed in Treatment 1 and 754 in Treatment 2, instead of the planned 500 in each. The time available for data collection was sufficient to meet the planned baseline target for Treatment 4.

The smaller-than-planned baseline sample sizes in the control area and Treatment 3 reduced the study’s ability to detect small changes in vaccination prevalence. To determine the minimum detectable change in Treatment 3, given its smaller baseline sample, we calculated the percentage-point increase required to achieve adequate power. With 44 respondents at baseline, 516 at follow-up, and a baseline HPV vaccination prevalence of 11.4%, the study had 80% power (two-sided α = 0.05) to detect a 14-percentage-point increase in vaccination (from 11.4% to 25.4%). In 2023, caregivers in Dhaka Division reported a 33% vaccination prevalence following the HPV vaccine introduction. Hence, detecting a meaningful change in Treatment 3 remained feasible despite the smaller-than-planned baseline sample.

The national campaign was implemented in October-November 2024 and the follow-up survey data was collected from 13/12/2024 to 23/12/2024. Baseline and follow-up surveys were conducted in a 1 km geofenced area around intervention and control schools using Meta’s audience targeting tools. For Treatment 4 (the population-wide social-media intervention), ads and survey recruitment were not limited to the 1 km geofenced areas but reached caregivers across entire divisions.

Participants were recruited via advertisements displayed on Facebook and Instagram.

Although schools were randomly assigned to treatment conditions, caregivers were recruited through Meta advertisements targeted geographically to the catchment areas of participating schools. Meta’s audience targeting tools allowed advertisements to be delivered to users located within defined geographic areas surrounding intervention schools. This approach ensured that respondents were likely to reside within the intervention areas where the social media campaigns were implemented. While perfect correspondence between school assignment and caregiver exposure cannot be guaranteed, geographic targeting allowed the survey sample to approximate the population of caregivers residing within the intervention areas.

Advertisements were designed to reach caregivers aged 18 and older in the six study divisions and described the study’s purpose. Respondents who identified themselves as caregivers of adolescent girls aged 9–17 were eligible to participate.

Upon clicking the advertisement, respondents were directed to a web-based survey within the Facebook app. The survey began with an introductory message explaining the study’s purpose, its approval by a Bangladeshi ethics board, and emphasized the voluntary nature of participation. It also provided compensation details. The consent process was approved by the IRB. Participants provided implied consent by clicking “start” to initiate the survey

Caregivers included mothers, fathers, uncles, aunts and other family members who were responsible for adolescent girls 9–17. At the start of the survey, respondents were asked “Are you the caregiver of a girl?” They were then asked about the age of the girl and their relationship to the girl. To ensure data privacy, all responses were anonymized. The survey instrument had been pre-tested and used in Bangladesh in 2023 [11]. The instrument collected data on caregiver reports of their adolescent’s age and vaccination status and their own socio-demographic characteristics. Caregivers who participated in the survey received a mobile credit incentive of 100 Bangladeshi Taka (USD 0.85).

### Data analysis

Univariate analyses were conducted to present frequency distributions of sample characteristics and vaccination outcomes across treatment and control groups at both baseline and follow-up. The primary outcome variable was the caregiver’s response to the question, “Has a girl-child in your care received an HPV vaccination?” If a caregiver responded in the affirmative, the child was considered vaccinated against HPV.

Bivariate analyses were performed to examine relationships between caregiver characteristics (age, education, gender, residence), adolescent age and vaccine uptake. Multivariate logistic regression was conducted to evaluate the effect of the social media and school-based awareness interventions on vaccination uptake, adjusting for differences in sample characteristics. Results are reported as adjusted odds ratios (aORs) and 95% confidence intervals (CIs). The data analysis was conducted using STATA version 14. Cluster-robust standard errors were estimated using the STATA cluster command, clustering observations at the division level to account for potential intra-cluster correlation among respondents residing in the same division, who may share exposure to similar campaign environments, media messaging, service delivery conditions, implementation contexts, and social norms related to vaccination [17]. A p-value of <0.05 was considered statistically significant.

Due to the implementation of the national HPV vaccination campaign across all six divisions of Bangladesh, we anticipated changes in vaccine uptake across all groups - including the control group. Thus, the analysis was designed to assess the additional effects of four interventions, over and above the effects of the national campaign – targeted social media and school awareness (T1), targeted social media alone (T2), school awareness alone (T3) and population–wide social media (T4). Data from the baseline and follow-up surveys were pooled to assess the impact of interventions in a context where rapid increases in HPV vaccination rates were expected.

#### Adjustment for baseline differences.

Socio-demographic differences between treatment and control groups were taken into account in the multivariate logistic regression analysis. Dummy variables for time (baseline = 0, follow-up = 1) and control and intervention groups (C, T1, T2, T3, T4) were included in the pooled analysis. Caregiver age, education, gender, urban or rural residence and the adolescent’s age, were included in the model to take differences in sample characteristics into account. Interaction terms (e.g., Time × T1) captured differential effects of interventions over time. Cluster-robust standard errors accounted for intra-cluster correlation.

#### Interaction effects analysis.

Interaction terms in the multivariate logistic regression model were used to assess whether the interventions had an impact on vaccine uptake. Each term (e.g., Time × T2) measured the additional effect of a specific intervention relative to the control group (C). A statistically significant interaction term indicated that an intervention was associated with an independent differential change in vaccination rates beyond that observed in the national campaign. For example, the likelihood of HPV vaccination increasing due to the targeted social media campaign alone (T2) is captured by the Time × T2 interaction term (OR = 1.92, 95% CI: 1.40–2.65, p < 0.001). In both models, odds ratios are adjusted for caregiver age, education, gender, urban or rural residence, and the adolescent girl’s age. In Model 2, main effects for sociodemographic covariates and intervention group are included in model estimation but are not displayed in the table for ease of reading.

### Study limitations

This study has several methodological limitations. First, recruitment through Meta social media advertisements may have excluded caregivers without internet or social media access, potentially over-representing younger, digitally connected, and urban caregivers and limiting generalizability to the broader caregiver population. Second, the unit of randomization (school) differs from the unit of analysis (individual caregiver), as caregivers self-selected into the survey via Meta advertisements rather than being enrolled through school clusters; cluster-robust standard errors were used to account for this discrepancy. Third, baseline sample sizes in the control group and Treatment 3 were smaller than planned due to the staggered release of Meta advertisement sets, reducing statistical power for detecting small changes in these groups, though the minimum detectable effect remained programmatically meaningful. Fourth, Treatment 4 was allocated at the division level rather than the school level, introducing potential geographic and contextual differences that school-level randomization did not control for. Fifth, adolescent vaccination status was reported by caregivers and was not independently verified through vaccination records, introducing the possibility of recall error or social desirability bias.

## Results

### HPV vaccination levels and caregiver characteristics

Baseline and follow-up HPV vaccination levels are shown in Table 3. In the Control Group (C), 10.1% of adolescent girls were vaccinated at baseline, increasing to 32.9% immediately after the national campaign. This represents a 22.8 percentage-point increase in the HPV vaccination rate. Similar or larger increases were observed in the intervention areas. A larger increase in the vaccination rate was observed in Treatment 1 (T1), where coverage rose by 26.3 percentage points (6.5% to 32.8%). Treatment 2 (T2) showed a 29.4 percentage point increase in HPV vaccination (6% to 35.4%). Treatment 3 (T3) showed an increase of 26 percentage points (from 11.4% to 37.4%). In Treatment 4, vaccination levels (T4) increased from 8.5% at baseline to 36.2% at follow-up, a 27.7 percentage-point increase. Although the national mass vaccination campaign was the primary contributor to these increases, larger increases in T1, T2, T3 and T4 compared to the control group suggest that these interventions played a meaningful role in increasing HPV vaccination rates.

#### Baseline characteristics of the Control Group (C).

The baseline characteristics of caregivers in the Control Group (C) provide a reference point for comparison with caregivers in the intervention groups (T1, T2, T3 and T4). In terms of caregivers’ ages, a majority (52.2%) were between 30 and 39 years, while 25.8% were 40 years or older, and 22.0% were ages 18–29. The gender distribution leaned towards a higher proportion of male caregivers (54.7%). Most caregivers had either higher secondary education (22.6%) or a bachelor’s degree (20.1%). Only 30.2% of caregivers had less than higher secondary education.

A majority of adolescent girls, 59.8%, were ages 9–11, followed by 27.0% ages 12–14, and 13.2% ages 15–17. About 64.2% of caregivers lived in urban areas, while 35.8% lived in rural areas.

#### Comparability of caregiver characteristics across groups.

While the characteristics of caregivers in the intervention groups (T1, T2, T3 and T4) were comparable to caregivers in the control group (C) at baseline, there were some differences. For example, at baseline, T3 had a higher proportion of male caregivers (65.4%) compared to C (54.7%), and C had a larger share of caregivers with master’s degrees (27.0%) than T1 (22.8%). Residence patterns also varied; C had a greater proportion of urban caregivers (64.2%) compared to T1 (54.7%). While most differences were not large, they highlighted the importance of adjusting for socio-demographic characteristics when estimating relative intervention effects. As a result, in logistic regression analyses to evaluate the relative effects of the four interventions, we adjusted for differences in sample characteristics at baseline and follow-up.

#### Implications for further analysis.

The substantial increases in vaccination levels across all groups suggest a broad effect of the national mass vaccination campaign. Somewhat larger relative increases observed in T1, T2, T3 and T4, suggested that these interventions had a measurable impact.

### Caregiver characteristics and their relationship to HPV vaccination

#### Overview.

Table 4 examines the relationships between caregivers’ characteristics and the likelihood of adolescent girls getting vaccinated against HPV. These findings help identify factors associated with vaccine uptake and inform the inclusion of variables in the multivariate analysis conducted to measure campaign effects presented in Table 5.

**Table 4:** Caregiver Characteristics and their Relationship to HPV Vaccination.

Variables	Adolescent Girls Vaccinated n (%)	P value
**Time**		
Baseline	293 (7.7)	<0.001
Follow-up	2461 (35.1)	
**Age of caregiver**		
18-29	1025 (27.0)	<0.001
30-39	930 (22.9)	
40 and older	799 (27.0)	
**Gender of caregiver**		
Male	1410 (24.4)	0.007
Female	1344 (26.6)	
**Education of caregiver**		
Less than higher secondary	830 (23.8)	0.046
Higher secondary	726 (25.9)	
Bachelor’s	573 (27.0)	
Master’s	625 (25.9)	
**Age of adolescent girl**		
9-11	1326 (21.0)	<0.001
12-14	1000 (35.7)	
15-17	428 (25.0)	
**Residence**		
Urban	1525 (25.5)	0.970
Rural	1229 (25.4)	
**Total**	2754 (25.4)	

**Table 5:** Multivariate Logistic Regression Models of HPV Vaccination. *Model 1: Main Effects; Model 2: Interaction Effects.*

Variables	(1) Main Effects	P value	(2) Interactions Only*	P value
**Time**				
Baseline	1.00			
Follow-up	6.56 (6.11-7.05)	<0.001		
**Intervention Type**				
C (National campaign only)	1.00			
T1 (National + targeted social media+ school awareness)	0.96 (0.91–1.02)	0.179		
T2 (National + targeted social media)	1.06 (0.99–1.13)	0.120		
T3 (National + school awareness)	1.20 (1.07–1.34)	0.002		
T4 (National + population-wide social media)	1.12 (0.97–1.31)	0.130		
**Age of caregiver**				
18-39	1.00			
40 and older	1.16 (1.09–1.24)	<0.001		
**Gender of caregiver**				
Male	1.00			
Female	1.14 (1.05–1.25)	0.004		
**Education of caregiver**				
Less than higher secondary	0.83 (0.74–0.94)	0.003		
Higher secondary or more	1.00			
**Age of adolescent girl**				
All other (9-11 & 15-17)	1.00			
12-14	1.94 (1.78-2.12)	<0.001		
**Residence**				
Urban (City, Town)	1.00			
Rural	1.10 (1.02-1.19)	0.013		
**Interactions**				
T1 x Follow-up			1.52 (1.30–1.77)	<0.001
T2 x Follow-up			1.92 (1.40–2.65)	<0.001
T3 x Follow-up			1.08 (1.01–1.16)	0.029
T4 x Follow-up			1.36 (1.28–1.45)	<0.001
**R-squared**	10.91%		10.95%	

**Note: For interactions, odds ratios greater than 1.0 indicate higher odds of HPV vaccination relative to the control group. All odds ratios are adjusted for caregiver age, education, gender, residence, and adolescent age. Main effects for sociodemographic covariates are included in Model 2 but not displayed.**

*For ease of reading, main effects are not shown for this model

Vaccination rates increased significantly between baseline and follow-up surveys. At baseline, 7.7% of adolescent girls were vaccinated, compared to 35.1% at follow-up (p < 0.001), indicating a substantial overall increase during the study period.

The proportion of adolescent girls vaccinated varied by caregiver’s age (p < 0.001). Vaccination rates were higher among girls with caregivers ages 18–29 (27.0%) and 30 and older (27.0%), while caregivers ages 30–39 had lower vaccination rates (22.9%).

A significant association was observed between caregivers’ gender and the adolescents’ vaccination status (p = 0.007). Adolescent girls who had female caregivers had higher vaccination rates (26.6%) compared to adolescent who had male caregivers (24.4%).

Vaccination rates were also associated with caregivers’ education level (p = 0.046). Adolescent girls whose caregivers had a bachelor’s degree had higher vaccination rates (27.0%) than those with higher secondary education (25.9%) or a master’s degree (25.9%). Girls whose caregivers who had less than higher secondary education had the lowest vaccination rates (23.8%).

Adolescent girls’ age was strongly associated with vaccination status (p < 0.001). Girls ages 12–14 had the highest vaccination rates (35.7%), followed by those aged 15–17 (25.0%). Vaccination rates were lowest among younger girls aged 9–11 (21.0%).

#### Implications for further analysis.

The significant associations observed between vaccination status and variables such as time, caregiver’s age, gender, education, and adolescent’s age highlight the importance of these factors in understanding HPV vaccine uptake. The findings highlighted the importance of including these variables in the multivariate analysis in Table 5, to adjust for potential confounding effects of these variables and to isolate the relative impact of the interventions.

### Logistic regression models for HPV vaccination

#### Main effects model (Model 1).

The main effects model in Table 5 explores the independent relationships between caregiver characteristics and vaccination status. HPV vaccination rates were significantly higher at follow-up compared to baseline (aOR = 6.56, 95% CI: 6.11–7.05; p < 0.001). This indicates that adolescent girls had a more than six times higher odds of being vaccinated at follow-up compared to baseline.

Caregivers ages 40 and older had 16% higher odds of reporting that adolescents in their care were vaccinated compared to caregivers ages 18–39 (aOR = 1.16, 95% CI: 1.09–1.24; p < 0.001). Female caregivers had significantly higher odds of getting their adolescent girls vaccinated compared to male caregivers (aOR = 1.14, 95% CI: 1.05–1.25; p = 0.004). Adolescents whose caregivers had less than higher secondary education had 17% lower odds of being vaccinated compared to those with higher education (aOR = 0.83, 95% CI: 0.74–0.94; p = 0.003). Adolescent girls aged 12–14 had nearly twice the odds of being vaccinated compared to those ages 9–11 or 15–17 (aOR = 1.94, 95% CI: 1.78–2.12; p < 0.001). Adolescents in rural areas had 10% higher odds of being vaccinated compared to those in urban areas (aOR = 1.10, 95% CI: 1.02–1.19; p = 0.013).

#### Interaction effects (Model 2).

The interaction model in Table 5 tests how the effect of time on vaccination rates differs by intervention type, offering insights into the relative impact of each intervention.

#### Targeted social media intervention + in-school awareness.

The targeted social media intervention with the in-school awareness intervention (T1) was associated with a significantly higher HPV vaccination rate compared to the control group (aOR = 1.52, 95% CI: 1.30–1.77; p < 0.001).

#### Targeted social media intervention alone.

The targeted social media intervention alone (T2) was associated with a significantly higher HPV vaccination rate compared to the control group (aOR = 1.92, 95% CI: 1.40–2.65; p < 0.001).

We tested for statistically significant differences between the magnitude of effect of the targeted social media intervention alone (aOR=1.92) and the targeted social media intervention combined with the in-school awareness intervention (aOR=1.52). There was no statistically significant difference between these two odds ratios (p = 0.1871, not shown) – indicating the lack of a statistically meaningful difference between T1 and T2.

#### School awareness intervention.

The school awareness intervention (T3) was associated with an 8% increase in the odds of vaccination compared to the control group (aOR = 1.08, 95% CI: 1.01–1.16; p = 0.029).

#### Population-level social media intervention.

The population-wide social media intervention (T4) was associated with a significant increase in the HPV vaccination rate compared to the control group (aOR = 1.36, 95% CI: 1.28–1.45; p < 0.001).

We tested whether there was any difference between the effects of the targeted social media intervention alone (aOR=1.92) and the population-wide social media intervention (aOR=1.36). There was a statistically significant difference between these two odds ratios (p < 0.001, not shown), indicating that the targeted social media intervention had a statistically stronger impact on vaccine uptake than the population-wide social media intervention.

To enhance interpretability for policy decisions, adjusted marginal effects (AMEs) were also estimated based on Model 2. The AMEs showed that, relative to the control group and after adjusting for socio-demographic characteristics, the probability of HPV vaccination increased by approximately 7.4 percentage points in Treatment 1, by 11.8 percentage points in Treatment 2, by 1.3 percentage points in Treatment 3, and by 5.3 percentage points in Treatment 4. These findings are consistent in both direction and significance with the odds ratios reported in Table 5.

## Discussion

The findings of this study show that the national introduction of the single-dose HPV vaccine in Bangladesh was associated with substantial increases in HPV vaccination rates across the country. The 22.8 percentage-point increase observed in the control group highlights the success of the government’s school-based vaccination program. The school-based delivery model likely contributed to reduced barriers to access, making it easier for caregivers to ensure their daughters were vaccinated. At the same time, with fewer than one-quarter of adolescents vaccinated at follow-up, considerable room remains for the national program to further increase HPV vaccination coverage among adolescent girls.

To measure the contribution of the social media and in-school awareness interventions to the national vaccination campaign, the study compared the rate of change in vaccination in the four intervention groups to the rate of change observed in the control group. Gains in vaccination rates were observed across all four interventions implemented to support the national program. Larger increases in vaccination rates were observed in areas where social media interventions were implemented. The largest contribution was observed in the targeted social media intervention, which translated into an approximately 12-percentage point increase in vaccination coverage. In comparison, the population-wide social media intervention contributed an approximately 5-percentage point increase in vaccination coverage. These findings suggest that social media interventions delivered in a targeted manner within the catchment areas of schools can have substantially larger impacts than those delivered broadly at the population level.

The stronger effects of targeted social media interventions have important implications for digital health strategies. Many studies examining vaccine demand in low- and middle-income countries report relatively high levels of caregiver motivation to vaccinate their children but lower levels of ability, reflecting barriers such as lack of information about vaccine availability, uncertainty about vaccination sites or limited access to services. In such contexts, broad digital messaging alone may have lower impact — individuals who lack the ability to act cannot easily translate motivation into behavior. However, when both motivation and ability are already high — such as when vaccination services are available nearby—social media messaging can serve as an effective behavioral prompt that encourages individuals to act. By targeting messages to communities where vaccination services were available in nearby schools, the intervention may have been particularly effective at prompting caregivers with existing motivation and ability to act. This finding is consistent with the Fogg Behavior Model, which posits that behavior occurs when motivation, ability, and prompts converge. From a programmatic perspective, the findings suggest that digital health campaigns may achieve greater impact when they are geographically targeted and coordinated with service delivery systems so that individuals have both the motivation and the practical ability to respond to campaign messages.

These findings also contribute to the growing literature on the role of social media metrics in public health communication. Social media platforms are increasingly recognized as important tools for disseminating health information and influencing health behavior in low- and middle-income countries. However, much of the existing literature evaluating digital health campaigns relies primarily on platform-generated metrics such as reach, impressions, likes, shares, or click-through rates. While these indicators provide useful information about message dissemination, they offer limited insight into whether audiences meaningfully engaged with the content or whether exposure translated into changes in attitudes, intentions, or behavior. By collecting survey data from individuals exposed to the campaign, this study provides a more direct way of examining how digital messaging relates to behavior. This approach demonstrates how digital surveys can be used to link exposure to social media interventions with individual-level behavior, offering a pathway for more rigorous evaluation of digital public health interventions.

Another contribution of this study is its application of a behavioral framework throughout the intervention process—from generating behavioral insights to informing intervention design and structuring the evaluation. In many public health programs, behavioral theories are used primarily to interpret findings after interventions have been implemented or are only loosely incorporated into program design. In contrast, this study used the same behavioral constructs to organize formative research, shape campaign messaging, and measure key drivers of behavior. Although the present analysis does not formally test the causal mechanisms linking campaign exposure to vaccination outcomes, the approach illustrates how practical behavioral models can provide a coherent structure for translating behavioral insights into scalable public health interventions while also enabling measurement of the changes in behavior.

Future research could extend this approach by explicitly testing mediation pathways to determine whether changes in motivation, ability, or perceived social norms explain the relationship between campaign exposure and vaccination behavior. In a forthcoming paper analyzing data from a related social media campaign implemented in Nigeria, we show that higher exposure to campaign messaging was associated with increases in caregivers’ motivation and ability to vaccinate their children. Conducting similar analyses in Bangladesh and other contexts would help determine whether these behavioral pathways operate consistently across settings and would strengthen the evidence base on how digital communication campaigns influence vaccination behavior.

### Scalability and cost-effectiveness of social media campaigns

A key strength of this study is its large-scale implementation across six divisions in Bangladesh. This demonstrates the feasibility of scaling social media-based interventions to support national public health efforts. Evidence from an economic evaluation of the Bangladesh campaign indicates that behavioral insights-informed social media interventions can be highly cost-effective. That analysis estimated an incremental cost of approximately $6 per additional vaccinated girl and about $40 per disability-adjusted life year (DALY) averted, well below commonly used cost-effectiveness thresholds for low- and middle-income countries [18]. These findings suggest that digital campaigns can represent an efficient strategy for increasing vaccine uptake when layered on top of national immunization programs.

At the same time, future research should explore the relative cost-effectiveness of targeted versus population-wide social media campaigns. While the present study finds that targeted social media interventions produced larger increases in vaccination rates, targeted campaigns may also require additional planning, geographic segmentation, and monitoring. Comparing the costs and impacts of targeted and population-wide strategies would help determine whether the additional effort and resources required for precision targeting yield sufficient returns in terms of increased vaccination coverage. Such analyses would provide important guidance for policymakers seeking to allocate resources efficiently when integrating digital demand generation strategies into national immunization programs.

### Why the school-based promotion intervention may have had a more modest effect

Although the school awareness intervention had a statistically significant effect, its impact on vaccination coverage was relatively modest, increasing the HPV vaccination rate by approximately 1.3 percentage points. One explanation may be that HPV vaccinations were already being administered in schools through the national program, leaving limited scope for additional gains from awareness activities conducted within the same setting. When vaccine delivery is already integrated into the school system, additional awareness-raising activities may have limited incremental impact on vaccination rates. Another possible explanation is that the school awareness intervention was not informed by the behavioral insights that guided the social media campaigns. The foundational behavioral insights data collected in 2023 were obtained from caregivers of adolescent girls rather than from the adolescents themselves. Collecting behavioral insights directly from adolescent girls may help design more effective in-school interventions by identifying ways to strengthen adolescents’ ability to communicate with caregivers about vaccination and to encourage caregivers to support HPV vaccination.

### Study limitations

This study has several limitations. First, while internet penetration has expanded rapidly in Bangladesh, recruitment through social media likely excluded caregivers without internet or social media access. As a result, the sample may over-represent younger, digitally connected, and urban caregivers, potentially limiting the generalizability of the findings to the broader caregiver population. However, because the social media interventions evaluated in this study were themselves delivered through digital platforms, recruiting respondents through the same platforms allowed the study to capture the population most likely to be exposed to the intervention.

Second, adolescent vaccination status was reported by caregivers and was not independently verified through vaccination records. As a result, responses may be subject to recall error or social desirability bias. Exposure to pro-vaccination messages during the social media campaign may have increased the likelihood that some caregivers reported vaccination in ways that aligned with perceived social expectations. At the same time, the national HPV vaccination campaign included mass media and interpersonal communication activities implemented by the government and its partners. This means that caregivers across both intervention and control areas were exposed to pro-vaccination messaging. Because the same survey instrument and recruitment procedures were used across all study groups at baseline and follow-up, any reporting bias would likely affect groups similarly and is unlikely to fully explain the differential intervention effects observed between intervention and control areas.

Third, the control group and the school awareness intervention group had smaller-than-planned baseline sample sizes because the Meta advertising algorithm released advertisement sets for these groups later than for other groups. This limited the time available for baseline data collection and reduced the number of caregivers who could be interviewed. Smaller baseline samples may reduce statistical power and increase uncertainty in estimates of baseline vaccination levels. However, substantially larger follow-up sample sizes were achieved for these groups, and the pooled regression analysis adjusted for socio-demographic characteristics and clustering within intervention areas. In addition, the distributions of key caregiver and adolescent characteristics were broadly similar across study groups at baseline, suggesting that the samples remained reasonably comparable.

## Conclusion

This study highlights the value of behavioral insights-informed interventions in supporting large-scale national vaccination campaigns. While the government’s school-based HPV vaccination campaign played a central role in increasing vaccination coverage, the additional gains observed in areas receiving social media interventions suggest the potential of digital strategiesto complement national public health efforts. As social media use continues to rise in low- and middle-income countries, leveraging digital platforms through interventions informed by behavioral insights and coordinated with service delivery systems may help convert existing caregiver motivation and ability into behavior, thereby strengthening immunization programs and improving vaccine uptake.

## Supporting information

S1 Checklist TREND Statement Checklist. (DOC)

## References

[1] World Health Organization. Bangladesh: Human papillomavirus and related cancers, fact sheet 2022. Geneva: WHO; 2022.

[2] Drolet M, Bénard É, Pérez N, et al. Population-level impact and herd effects following HPV vaccination programs: A systematic review and meta-analysis. Lancet Infect Dis. 2019;19(3):e171–80. 10.1016/S1473-3099(14)71073-4 PMC5144106 25744474

[3] Uddin J, Shamsuzzaman M, Horng L, et al. Evaluation of the impact of the measles-rubella campaign on vaccination coverage and routine immunization services in Bangladesh. Vaccine. 2016;34(32):3744–51. 10.1186/s12879-016-1758-x PMC4983043 27519586

[4] Thangaraj JWV, Prosperi C, Kumar MS, et al. Post-campaign coverage evaluation of a measles and rubella supplementary immunization activity in five districts in India, 2019-2020. PLoS One. 2024;19(3):e0297385. doi: 10.1371/journal.pone.0297385 PMC10980234 38551928

[5] Danovaro-Holliday MC, Rhoda DA, Lacoul M, Prier ML, Gautam JS, Pokhrel TN, et al. Who gets vaccinated in a measles-rubella campaign in Nepal?: results from a post-campaign coverage survey. BMC Public Health. 2022;22(1):221. doi: 10.1186/s12889-021-12475-0 35114969 PMC8812357

[6] Ogunniyi TJ, Rufai BO, Uketeh SN, Turzin JK, Oyinloye EA, Effiong FB. Two years of COVID-19 vaccination in Nigeria: a review of the current situation of the pandemic: a literature review. Ann Med Surg (Lond). 2023;85(11):5528–32. doi: 10.1097/MS9.0000000000001310 37915653 PMC10617854

[7] Mone Hardy CM, Rathee M, Chaudhury S, et al. Progress toward poliomyelitis eradication—Afghanistan, January 2023–September 2024. MMWR Morb Mortal Wkly Rep. 2024;73(49):1129–34. 39666643 10.15585/mmwr.mm7349a4 PMC11637418

[8] Centers for Disease Control and Prevention (CDC). Progress toward poliomyelitis eradication—Pakistan, January 2023–June 2024. MMWR Morb Mortal Wkly Rep. 2024;73(36):788–92. 39264848 10.15585/mmwr.mm7336a2 PMC11392225

[9] Umutesi G, Oluoch L, Weiner BJ, Bukusi E, Onono M, Njoroge B, et al. HPV vaccination in Kenya: a study protocol to assess stakeholders’ perspectives on implementation drivers of HPV vaccination and the acceptability of the reduced dose strategy among providers. Front Health Serv. 2023;3:1233923. doi: 10.3389/frhs.2023.1233923 37600926 PMC10433907

[10] Binagwaho A, Wagner C, Gatera M, Karema C, Nutt C, Ngaboa F. Achieving high coverage in Rwanda’s national human papillomavirus vaccination programme. Bull World Health Org. 2012;90(8):623–8. doi: 10.2471/blt.11.097253 22893746 PMC3417784

[11] Agha S, Francis S, Bernard D, et al. Effects of a multimedia campaign to increase HPV vaccine acceptance in Dhaka, Bangladesh. Hum Vaccin Immunother. 2025;21(1):e2447105. 10.1080/21645515.2024.2447105 PMC11730613 39780523

[12] Noreen K, Naeem Khalid S, Murad MA, Baig M, Khan SA. Uptake and determinants of HPV vaccination in South Asia: a systematic review and meta-analysis. Front Public Health. 2024;12:1453704. doi: 10.3389/fpubh.2024.1453704 39722717 PMC11668735

[13] Junaidi I. Only 34pc of HPV vaccination target achieved as drive nears end. *Dawn*. 2025. Available from: https://www.dawn.com/news/1944376/only-34pc-of-hpv-vaccination-target-achieved-as-drive-nears-end

[14] Lubeya MK, Chibwesha CJ, Mwanahamuntu M, et al. Determinants of implementation of HPV vaccination in Zambia: Application of the CFIR framework. Vaccines. 2024;12(1):32. 10.3390/vaccines12010032 PMC10821054 38250845

[15] World Health Organization (WHO). Post-introduction evaluation of HPV vaccination in Gazipur, Bangladesh. Geneva: World Health Organization; 2017. Available from: https://www.who.int/bangladesh/news/detail/15-01-2017-post-introduction-evaluation-of-human-papilloma-virus-vaccination-held-in-gazipur

[16] Fogg BJ. A behavior model for persuasive design. Proceedings of the 4th International Conference on Persuasive Technology (Article No. 40). Claremont (CA): Association for Computing Machinery; 2009. p. 1–7.

[17] StataCorp. Stata release 15: Statistical software. College Station (TX): StataCorp LLC; 2017.

[18] Agha S, Zeng W. Cost-effectiveness of a behavioral insights-informed digital campaign to increase HPV vaccination in Bangladesh. Hum Vaccin Immunother. 2025;21(1):2500264. doi: 10.1080/21645515.2025.2500264 40322786 PMC12054372

