# Supplementary material for: Impact of behavioral insights-informed social media campaigns on HPV vaccination in Bangladesh
Source: PLOS Glob Public Health. 2026 Jun 23;6(6):e0006732. doi: 10.1371/journal.pgph.0006732 (PMC13289952; doi:10.1371/journal.pgph.0006732)
Supplement: Attachment — Submitted filename: Comments_PGPH-D-26-00063.doc [file pgph.0006732.s002.doc]

This manuscript addresses a very important public health concern. It integrates behavioral insights in digital strategies. It is innovative and responds to the current needs. Some comments are highlighted to improve the quality and robustness of the paper.

Large discrepancies between the different sample sizes which would affect the accuracy and validity of the analysis. This should be carefully addressed.

The recruitment through meta advertisement differs from the schools-based sample frame. This leads to selection bias and discrepancy between school assignment and caregiver exposure. Explain and justify.

Self-reported vaccination outcomes could be biased due to exposure to social media messages.

Selection bias as findings relate to a population that has access to digital resources.

Some important details about the experiment are missing like Impressions per user, Frequency of exposure, duration of the campaign, etc.

Explain the cluster-robust standard errors and specify the categories and numbers.

In summary, the experiment should be clearly described and limitations controlled and justified.

Make the tables easier to read.

Revise the discussion section, provide deeper interpretation of findings, and link them more comprehensively to the broader contextual and global literature.

Reconsider the limitations of the study in light of the above comments.
