# Supplementary material for: Impact of behavioral insights-informed social media campaigns on HPV vaccination in Bangladesh
Source: PLOS Glob Public Health. 2026 Jun 23;6(6):e0006732. doi: 10.1371/journal.pgph.0006732 (PMC13289952; doi:10.1371/journal.pgph.0006732)
Supplement: Attachment — Submitted filename: 2026 3 11 Response to Editor and Reviewer Comments.docx [file pgph.0006732.s003.docx]

**Response to Editor and Reviewer Comments**

Dear Editor and Reviewer,

We thank the editor and reviewer for the careful reading of our manuscript and for the constructive comments. We appreciate the positive assessment that the study addresses an important public health concern and integrates behavioral insights into digital strategies. We have revised the manuscript substantially to address the comments and to improve the clarity, rigor, and interpretation of the study. Below we respond to each comment in detail.

**Editor Comments and Author Responses**

**Comment 1 – Ethics statement location**

Please ensure that your Ethics Statement is available in its entirety at the beginning of your Methods section, under a subheading “Ethics Statement”.

**Response:**
We have moved the ethics statement to the beginning of the Methods section under the subheading **“Ethics Statement”**, as requested. The section now clearly presents the ethical approval information and the consent procedures used in the study.

**Comment 2 – Financial disclosure statement**

Please amend your detailed Financial Disclosure statement and clarify all sources of financial support for your study.

**Response:**
The Financial Disclosure statement has been revised to clearly identify the funding source, grant number, grant recipient, and salary support for the author. The revised statement also clarifies the role of the funders in the study. The Financial Disclosure now reads as follows:

This work was supported by the Bill & Melinda Gates Foundation [INV-061346] grant titled “Leveraging Social Media for Messaging HPV Vaccine Uptake in Bangladesh,” awarded to Upswell LLC. Author SA (Sohail Agha) received salary support from Upswell LLC during the conduct of the study. The author’s time was also supported by the Behavioral Insights Lab. The funders had no role in study design, data analysis, decision to publish, or preparation of the manuscript.

**Reviewer Comments and Author Responses**

**Comment 1**

Large discrepancies between the different sample sizes which would affect the accuracy and validity of the analysis. This should be carefully addressed.

**Response:**
We appreciate the reviewer’s attention to this issue. The discrepancies in baseline sample sizes occurred because Meta’s advertising algorithm released advertisement sets for some intervention groups later than others, reducing the available time for baseline recruitment in those groups. This issue particularly affected the control group and the school-awareness intervention group.

To address potential concerns about statistical validity, we have clarified this issue in the manuscript and explained the steps taken to mitigate its impact. First, substantially larger follow-up sample sizes were obtained for these groups. Second, the regression analysis adjusts for socio-demographic characteristics and uses cluster-robust standard errors to account for clustering within intervention areas. Third, the distribution of key caregiver and adolescent characteristics was broadly similar across groups at baseline, suggesting that the samples remained reasonably comparable.

**Changes made:**
Clarification added to the Study Limitations section describing the baseline sample size differences and how they were addressed in the analysis.

**Comment 2**

The recruitment through Meta advertisement differs from the schools-based sample frame. This leads to selection bias and discrepancy between school assignment and caregiver exposure. Explain and justify.

**Response:**
We thank the reviewer for raising this important point. The recruitment strategy intentionally used the same digital platforms through which the intervention messages were delivered. This allowed the study to capture respondents who were most likely to be exposed to the social media campaign.

While this approach introduces the possibility of selection bias—particularly toward caregivers who have access to digital platforms—it also improves the ability to evaluate the intervention among the population actually exposed to the campaign. Because the intervention itself was delivered through social media, evaluating it within a digitally connected population provides a realistic assessment of its effectiveness.

We now explain this rationale more clearly in the manuscript and acknowledge the potential selection bias in the limitations section.

**Changes made:**
Expanded explanation in the Methods and Limitations sections.

**Comment 3**

Self-reported vaccination outcomes could be biased due to exposure to social media messages.

**Response:**
We agree that self-reported vaccination status may introduce recall or social desirability bias. We have clarified this limitation in the manuscript. However, several factors reduce the likelihood that this bias explains the observed intervention effects. First, the national HPV vaccination campaign included mass media and interpersonal communication activities implemented by the government, meaning that caregivers in both intervention and control areas were exposed to pro-vaccination messaging. Second, the same survey instrument and recruitment procedures were used across all groups at baseline and follow-up, making systematic reporting bias across groups less likely.

These clarifications have been added to the limitations section.

**Changes made:**
Expanded discussion of potential reporting bias in the Study Limitations section.

**Comment 4**

Selection bias as findings relate to a population that has access to digital resources.

**Response:**
We agree that the study population represents caregivers who have access to digital platforms and social media. This is now explicitly acknowledged as a limitation of the study. At the same time, because the intervention itself was delivered through digital channels, evaluating outcomes among digitally connected caregivers provides important insights into the effectiveness of digital public health strategies. As internet and smartphone access continues to expand in LMICs, understanding how digital interventions influence health behaviors among connected populations is increasingly important.

**Changes made:**
Clarified in the Study Limitations section.

**Comment 5**

Some important details about the experiment are missing like impressions per user, frequency of exposure, duration of the campaign, etc.

**Response:**
We thank the reviewer for this suggestion. Additional information about the campaign implementation has been added to the Methods section, including the duration of the campaign and the general approach to digital advertisement delivery. While individual-level exposure frequency cannot be measured precisely due to the nature of platform-based advertising algorithms, we now clarify how the campaign was implemented and monitored using Meta Ads Manager metrics.

**Changes made:**
Additional campaign implementation details added to the Methods section.

**Comment 6**

Explain the cluster-robust standard errors and specify the categories and numbers.

**Response:**
Thank you for this helpful comment. The manuscript now clarifies that cluster-robust standard errors were used to account for clustering within intervention areas. The rationale for clustering has been clarified in the statistical analysis section.

**Changes made:**
Additional explanation added to the Statistical Analysis section.

**Comment 7**

In summary, the experiment should be clearly described and limitations controlled and justified.

**Response:**
We appreciate this suggestion and have revised several sections of the manuscript to improve clarity. Specifically, we expanded the Methods section to better describe the intervention design and implementation, and we substantially revised the Limitations section to more explicitly address potential sources of bias, including selection bias, reporting bias, and sample size differences.

**Comment 8**

Make the tables easier to read.

**Response:**
The tables have been made easier to read. Non-essential information in Table 3 has been removed. A note has been added to Table 5 to make interpretation of interaction terms easier to understand.

**Changes made:**
Tables revised for improved readability.

**Comment 9**

Revise the discussion section, provide deeper interpretation of findings, and link them more comprehensively to the broader contextual and global literature.

**Response:**
We thank the reviewer for this valuable suggestion. The Discussion section has been substantially revised to provide deeper interpretation of the findings and to situate them within the broader literature on digital health and vaccination programs.

The revised discussion now highlights several contributions of the study, including:

• evidence that targeted social media campaigns can produce larger effects than population-wide campaigns, particularly when services are locally available
• interpretation of the findings using the Fogg Behavior Model, emphasizing the interaction between motivation, ability, and prompts
• the importance of aligning digital messaging with service availability
• the use of survey-based evaluation methods to measure behavioral outcomes rather than relying solely on social media platform metrics
• the scalability and cost-effectiveness of behavioral insights-informed digital campaigns

**Comment 10**

Reconsider the limitations of the study in light of the above comments.

**Response:**
The Limitations section has been revised and expanded to address all concerns raised by the reviewer. The revised section now discusses:

• selection bias due to digital recruitment
• potential reporting bias from self-reported vaccination status
• baseline sample size differences across groups
• limitations in measuring individual exposure to digital messaging

These revisions provide a more transparent and comprehensive discussion of the study’s limitations.

**Conclusion**

We are grateful for the editor’s and reviewer’s constructive feedback. The revisions have improved the clarity, rigor, and interpretation of the manuscript. We believe the revised version addresses the concerns raised and strengthens the paper’s contribution to the literature on digital health interventions and vaccination programs.

Thank you again for the opportunity to revise this manuscript.

Sincerely,
**Sohail Agha**
