# Supplementary material for: Impact of behavioral insights-informed social media campaigns on HPV vaccination in Bangladesh
Source: PLOS Glob Public Health. 2026 Jun 23;6(6):e0006732. doi: 10.1371/journal.pgph.0006732 (PMC13289952; doi:10.1371/journal.pgph.0006732)
Supplement: Attachment — Submitted filename: PLOS review.docx [file pgph.0006732.s004.docx]

**PLOS Global Public Health Impact of Behavioral Insights-Informed Social Media Campaigns on HPV Vaccination in Bangladesh --Manuscript Draft**

Recommendations for edits:

1. Study Design, page 5: As mentioned earlier, the national campaign was initiated In Dhaka Division in 202311

Suggestion: This sentence the red highlighted should be small “i” I guess

2. Page 7: The social media campaign focused on three key themes HPV vaccination enables a brighter future by protecting girls’ health.

Suggestion: This sentence is run on. Please insert colon (:) punctuation mark after… three key themes

Questions:

3. Is there any information on the influence of religion and caste on the study?
4. Did the study evaluate the impact of more men receiving social media interventions and uptake of HPV vaccination? Given men have a likely greater role in decision making.
