# Supplementary material for: Impact of behavioral insights-informed social media campaigns on HPV vaccination in Bangladesh
Source: PLOS Glob Public Health. 2026 Jun 23;6(6):e0006732. doi: 10.1371/journal.pgph.0006732 (PMC13289952; doi:10.1371/journal.pgph.0006732)
Supplement: Attachment — Submitted filename: PLOS Impact of Behavioral Insights - Reviewers Comments.docx [file pgph.0006732.s005.docx]

**Impact of Behavioral Insights-Informed Social Media Campaigns on HPV Vaccination in Bangladesh--Manuscript Draft**

**Strengths:**

1. Good choice of topic and relevant to address maternal health challenges in particular Cervical Cancer which is rampart
2. The background is well beefed up with relevant reference and similar findings in Asia, and other parts of the world

**Areas of Improvement:**

The methods section requires some improvement to enhance flow of ideas and help the reader to easily comprehend.

Pg 4: Concept of Treatment (T1, T2, T3, T4) appearing for the first time – could it be expanded. Are these treatment arms or study sites or refers to the campaign type?

Pg 8: “*The HPV vaccine was introduced in these divisions in October-November 2024*.” This cannot be under sampling section – remove

Pg 10: Sampling and Sampling Strategy – I recommend narrative regarding data collection to be place under the specific sub-section on Data collection. The author could also remove narrative on limitation and place under specific sub-section on Study Limitation.

Under Methods section – add study limitations
