# Supplementary material for: Impact of behavioral insights-informed social media campaigns on HPV vaccination in Bangladesh
Source: PLOS Glob Public Health. 2026 Jun 23;6(6):e0006732. doi: 10.1371/journal.pgph.0006732 (PMC13289952; doi:10.1371/journal.pgph.0006732)
Supplement: Attachment — Submitted filename: Response_to_Reviewers.docx [file pgph.0006732.s006.docx]

# Response to Reviewers

**Manuscript: Impact of Behavioral Insights-Informed Social Media Campaigns on HPV Vaccination in Bangladesh**

**Journal: PLOS Global Public Health**

**Manuscript ID: PGPH-D-26-00063R1**

We thank the editor and both reviewers for their careful reading of the manuscript and their constructive comments. We have addressed each point raised and believe the revisions substantially strengthen the manuscript. All changes are detailed below, with corresponding revisions highlighted in the tracked-changes version of the manuscript.

# Reviewer 1 (Dennis Cherian)

**Strengths noted by Reviewer 1:**

The reviewer noted the good choice of topic and relevance to maternal health challenges, particularly cervical cancer, and commended the well-referenced background section.

## Comment 1: Treatment arms (T1–T4) not clearly explained on first appearance (Page 4)

***Reviewer comment:*** *Concept of Treatment (T1, T2, T3, T4) appearing for the first time – could it be expanded. Are these treatment arms or study sites or refers to the campaign type?*

**Response:** We have added a clarifying sentence immediately after the list of four interventions in the Introduction, making explicit that T1–T4 denote distinct treatment arms (campaign types), not study sites. We have also corrected the order of the interventions in this paragraph so that it is consistent with the order used throughout the rest of the paper. The revised text reads:

*“Four interventions were implemented in coordination with the HPV vaccine introduction to increase the impact of the national campaign: (1) a targeted social media campaign combined with an in-school awareness campaign, (2) a targeted social media campaign alone, (3) a school awareness campaign alone, and (4) a population-wide social media campaign. These four interventions are referred to throughout this paper as treatment arms: Treatment 1 (T1), Treatment 2 (T2), Treatment 3 (T3), and Treatment 4 (T4), where each label denotes a distinct intervention type.”*

## Comment 2: Misplaced sentence in sampling section (Page 8)

***Reviewer comment:*** *"The HPV vaccine was introduced in these divisions in October-November 2024." This cannot be under sampling section – remove.*

**Response:** This sentence has been deleted from the Study population and sampling strategy subsection. The timing of the national campaign is already stated in the Data collection subsection (“The national campaign was implemented in October–November 2024”) and does not need to appear in the sampling section.

## Comment 3: Data collection narrative misplaced in sampling section (Page 10)

***Reviewer comment:*** *Sampling and Sampling Strategy – I recommend narrative regarding data collection to be placed under the specific sub-section on Data collection. The author could also remove narrative on limitation and place under specific sub-section on Study Limitation.*

**Response:** We agree. The following paragraphs have been moved from the Study population and sampling strategy subsection to the Data collection subsection, where they now appear after the opening sentence on survey dates:

(1) The paragraph describing the Meta algorithm’s staggered release of ad sets and its consequences for baseline sample sizes in the control group and Treatment 3.

(2) The paragraph describing the analytical approach used to address unequal baseline sample sizes.

(3) The paragraph on oversampling in Treatment 1 and Treatment 2.

(4) The paragraph on the minimum detectable change for Treatment 3 given its smaller baseline sample.

The Study population and sampling strategy subsection now contains only content directly related to the study population, stratified sampling approach, planned sample sizes, and power calculations.

## Comment 4: Add study limitations under Methods section

***Reviewer comment:*** *Under Methods section – add study limitations.*

**Response:** A Study Limitations subsection has been added at the end of the Methods section, after the Data analysis subsection. This subsection provides a concise methods-level summary of key limitations, consistent with TREND reporting guidelines. The detailed discussion of limitations in the Discussion section remains unchanged. The new subsection reads:

***Study Limitations***

*This study has several methodological limitations. First, recruitment through Meta social media advertisements may have excluded caregivers without internet or social media access, potentially over-representing younger, digitally connected, and urban caregivers and limiting generalizability to the broader caregiver population. Second, the unit of randomization (school) differs from the unit of analysis (individual caregiver), as caregivers self-selected into the survey via Meta advertisements rather than being enrolled through school clusters; cluster-robust standard errors were used to account for this discrepancy. Third, baseline sample sizes in the control group and Treatment 3 were smaller than planned due to the staggered release of Meta advertisement sets, reducing statistical power for detecting small changes in these groups, though the minimum detectable effect remained programmatically meaningful. Fourth, Treatment 4 was allocated at the division level rather than the school level, introducing potential geographic and contextual differences that school-level randomization did not control for. Fifth, adolescent vaccination status was reported by caregivers and was not independently verified through vaccination records, introducing the possibility of recall error or social desirability bias.*

# Reviewer 2 (Abanoub Riad)

## Comment 1: Reporting guideline and cluster-randomization clarification

***Reviewer comment:*** *The manuscript should be revised to follow an appropriate reporting guideline. Given that the study is presented as a pre-test/post-test quasi-experimental evaluation of behavioural and public health interventions, the TREND statement appears to be the most appropriate primary reporting framework. The authors should provide a completed TREND checklist and revise the manuscript accordingly. In addition, because the manuscript states that schools in some divisions were randomly assigned to intervention/control conditions, the authors should clarify whether the study includes a cluster-randomised component and, if so, address the relevant CONSORT cluster-trial reporting items. At present, the study design is insufficiently transparent: it is unclear whether the causal claims rest on non-randomised quasi-experimental comparisons, school-level randomisation, division-level allocation, or a combination of these.*

**Response:** We thank the reviewer for this important methodological point. We address both elements of the comment in turn.

### TREND Checklist

We agree that the TREND statement is the appropriate reporting framework for this study, given its pre-test/post-test quasi-experimental design evaluating behavioral and public health interventions in a non-randomized setting. A completed TREND checklist has been prepared and is submitted as a supplementary file accompanying this revision. All 22 items have been addressed, with page references provided for each reported element.

### Clarification of study design: quasi-experimental with partial school-level randomization

We appreciate the reviewer’s request for greater transparency on this point, and we agree the original manuscript did not state this clearly enough. We have added a dedicated paragraph to the Study Design section clarifying the design structure. In summary:

The study incorporates a school-level randomization component — public schools in Barisal, Chittagong, and Sylhet divisions were randomly assigned to treatment conditions (T1, T2, T3, or control). However, the study is correctly characterized as quasi-experimental rather than a cluster-randomized trial for the following reasons:

**1. Caregivers were not enrolled through the clusters.** Although schools were randomly assigned to treatment conditions, caregivers were recruited independently via Meta advertisements targeted geographically to the catchment areas of participating schools. Caregiver participation was determined by self-selection — clicking on a Facebook or Instagram advertisement — rather than by cluster membership. This means the unit of randomization (school) and the unit of recruitment (individual caregiver) are distinct, and caregiver assignment to treatment conditions was not controlled.

**2. The design is repeated cross-sectional, not a panel.** Baseline and follow-up respondents are not the same individuals, unlike a true cluster-randomized trial where the same participants are followed over time.

**3. Treatment 4 was allocated at the division level,** not the school level, representing a different and non-randomized allocation mechanism from T1–T3. The three divisions receiving T4 were distinct from the three divisions containing the control and other treatment groups, introducing potential geographic and contextual confounders that school-level randomization did not control for.

**4. Self-selection bias.** Caregivers who chose to click on Meta advertisements and complete the survey may differ systematically from those who did not, in ways that cannot be fully controlled even with covariate adjustment.

For these reasons, CONSORT cluster-trial reporting items do not fully apply to this design. The appropriate framework is the TREND statement, which is designed precisely for non-randomized evaluations of behavioral interventions. That said, we have reviewed the CONSORT cluster-trial extension items and addressed those that are relevant — specifically, we clarify the unit of assignment (school), the unit of analysis (individual caregiver), and the analytical method used to account for the discrepancy between these two units (cluster-robust standard errors clustered at the division level).

We have revised the Study Design section to make all of the above explicit. The revised paragraph reads:

*“The quasi-experimental design consisted of four intervention groups and one control group implemented across six divisions, allowing comparison of targeted social media, population-wide social media, and school-based awareness interventions relative to areas exposed only to the national campaign. Although schools in Barisal, Chittagong, and Sylhet divisions were randomly assigned to treatment conditions, the study is characterized as quasi-experimental rather than a cluster-randomized trial for several reasons. First, caregivers were not enrolled through the school clusters — they were recruited independently via Meta advertisements targeted geographically to the catchment areas of participating schools. Caregiver participation was determined by self-selection, meaning that the unit of randomization (school) and the unit of recruitment and analysis (individual caregiver) are distinct. Second, the design is repeated cross-sectional: baseline and follow-up respondents are not the same individuals, unlike a true cluster-randomized trial in which the same participants are followed over time. Third, Treatment 4 was allocated at the division level rather than the school level, representing a different and non-randomized allocation mechanism from T1–T3; the three divisions receiving T4 were geographically distinct from the three divisions containing T1–T3 and the control group, introducing potential contextual differences that school-level randomization did not control for. To account for the discrepancy between the unit of assignment (school) and the unit of analysis (individual caregiver), cluster-robust standard errors were estimated, clustering observations at the division level.”*

## Comment 2: Moderate causal language throughout the manuscript

***Reviewer comment:*** *The authors should moderate causal language throughout the manuscript. The study can support evidence of differential pre-post changes associated with intervention areas, but the current wording sometimes implies stronger causal attribution than the design and exposure measurement can support.*

**Response:** We agree. We have carefully reviewed the manuscript and moderated causal language throughout. The specific changes made are listed below.

**Abstract — Results paragraph**

**Original:** *HPV vaccination coverage increased from 10.1% to 32.9% in the control group, reflecting the effect of the national campaign.*

**Revised:** *HPV vaccination coverage increased from 10.1% to 32.9% in the control group, consistent with the national campaign being implemented across all study areas.*

**Abstract — Conclusions**

**Original:** *The targeted social media intervention had a significantly larger impact than the population-wide social media intervention.*

**Revised:** *The targeted social media intervention was associated with significantly larger increases in vaccination coverage than the population-wide social media intervention.*

**Results — Interaction effects, T1**

**Original:** *The targeted social media intervention with the in-school awareness intervention (T1) significantly increased the HPV vaccination rate compared to the control group.*

**Revised:** *The targeted social media intervention with the in-school awareness intervention (T1) was associated with a significantly higher HPV vaccination rate compared to the control group.*

**Results — Interaction effects, T2**

**Original:** *The targeted social media intervention alone (T2) significantly increased the HPV vaccination rate compared to the control group.*

**Revised:** *The targeted social media intervention alone (T2) was associated with a significantly higher HPV vaccination rate compared to the control group.*

**Discussion — Opening paragraph**

**Original:** *The findings of this study show that the national introduction of the single-dose HPV vaccine in Bangladesh resulted in substantial increases in HPV vaccination rates across the country.*

**Revised:** *The findings of this study show that the national introduction of the single-dose HPV vaccine in Bangladesh was associated with substantial increases in HPV vaccination rates across the country.*

**Discussion — Opening paragraph**

**Original:** *By administering vaccines directly in educational institutions, the national campaign reduced barriers to access and made it easier for caregivers to ensure their daughters were vaccinated.*

**Revised:** *The school-based delivery model likely contributed to reduced barriers to access, making it easier for caregivers to ensure their daughters were vaccinated.*

**Discussion — Fogg Behavior Model paragraph**

**Original:** *the intervention may have been particularly effective at converting existing motivation and ability into action.*

**Revised:** *the intervention may have been particularly effective at prompting caregivers with existing motivation and ability to act.*

**Data Analysis section**

**Original:** *A statistically significant interaction term indicated that an intervention had an independent effect beyond the national campaign.*

**Revised:** *A statistically significant interaction term indicated that an intervention was associated with an independent differential change in vaccination rates beyond that observed in the national campaign.*

**Conclusions**

**Original:** *the additional gains observed in areas receiving social media interventions demonstrate the potential of digital strategies.*

**Revised:** *the additional gains observed in areas receiving social media interventions suggest the potential of digital strategies.*

Language that was already appropriately hedged — including “associated with,” “suggest,” and standard statistical terminology such as “interaction effects” — has been retained unchanged.

## Comment 3: Regression analysis clarification and Table 5 footnote

***Reviewer comment:*** *The regression analysis requires substantial clarification and possibly re-analysis. It is not clear whether odds ratios of “Time”, “Intervention Type” and “Interactions” were adjusted for baseline characteristics or not. It is also unclear whether any of the baseline characteristics were adjusted. This footnote is unclear too: “Note: For interactions, odds ratios greater than indicate higher odds of HPV vaccination relative to the control group.”*

**Response:** We thank the reviewer for these important clarifications. We address each point in turn.

### Whether odds ratios are adjusted for baseline characteristics

We agree the original manuscript was insufficiently clear on this point. To clarify:

**Model 1 (Main Effects)** presents adjusted odds ratios. The model includes dummy variables for time (baseline = 0, follow-up = 1), intervention group (C, T1, T2, T3, T4), and the following sociodemographic covariates: caregiver age, caregiver education, caregiver gender, urban/rural residence, and adolescent age. All odds ratios in Model 1 are therefore adjusted for these baseline characteristics.

**Model 2 (Interaction Effects)** also includes the same sociodemographic covariates as Model 1, with the addition of interaction terms (Time × T1, Time × T2, Time × T3, Time × T4) to capture differential pre-post changes associated with each intervention relative to the control group. The interaction term odds ratios reported in Model 2 are therefore also adjusted for baseline sociodemographic characteristics. For ease of reading, the main effects coefficients are not displayed in Model 2, but they are included in the model estimation.

The following sentence has been added to the Data Analysis section, at the end of the Interaction effects analysis subsection:

*“In both models, odds ratios are adjusted for caregiver age, education, gender, urban or rural residence, and the adolescent girl’s age. In Model 2, main effects for sociodemographic covariates and intervention group are included in model estimation but are not displayed in the table for ease of reading.”*

### Table 5 footnote — missing value

The reviewer correctly identified that the Table 5 footnote contained an incomplete sentence. The value “1.0” was inadvertently omitted. The corrected footnote reads:

*“Note: For interactions, odds ratios greater than 1.0 indicate higher odds of HPV vaccination relative to the control group. All odds ratios are adjusted for caregiver age, education, gender, residence, and adolescent age. Main effects for sociodemographic covariates are included in Model 2 but not displayed.”*

# Minor Editorial Comments (PLOS Review Document)

## Comment 1: Capitalization error (Page 5)

**Original:** *"As mentioned earlier, the national campaign was initiated In Dhaka Division in 2023"*

**Revised:** *"As mentioned earlier, the national campaign was initiated in Dhaka Division in 2023"*

## Comment 2: Run-on sentence, missing colon (Page 7)

**Original:** *"The social media campaign focused on three key themes HPV vaccination enables a brighter future by protecting girls’ health."*

**Revised:** *"The social media campaign focused on three key themes: HPV vaccination enables a brighter future by protecting girls’ health."*

## Comments 3 and 4: Questions on religion/caste and male caregiver exposure

**Response:** We thank the reviewer for these questions. The study did not collect data on religion or caste, and given that Bangladesh is predominantly Muslim with limited religious variation in the study population, these variables were not considered analytically relevant to the study objectives. The role of caregiver gender in vaccination uptake is addressed through the multivariate analysis, which found that female caregivers had significantly higher odds of vaccination (aOR = 1.14, p = 0.004).

# Additional Correction

**Sample size description (Methods — Study population and sampling strategy):**

During the revision process, we identified an inconsistency in the description of the planned follow-up sample size. The original text stated “1,000 respondents each for treatment and control groups,” which did not accurately reflect the planned sample sizes for Treatment 3 (500) and Treatment 4 (3,000) shown in Table 2. The sentence has been corrected to read:

*“For the follow-up survey, we planned for larger sample sizes to compensate for smaller sample sizes at baseline: 1,000 respondents each in the control group, Treatment 1, and Treatment 2; 500 in Treatment 3; and 3,000 in Treatment 4, for a total planned follow-up sample of 6,500 caregivers.”*
