# Supplementary material for: Impact of behavioral insights-informed social media campaigns on HPV vaccination in Bangladesh
Source: PLOS Glob Public Health. 2026 Jun 23;6(6):e0006732. doi: 10.1371/journal.pgph.0006732 (PMC13289952; doi:10.1371/journal.pgph.0006732)
Supplement: Attachment — Submitted filename: HPV Vaccination in Bangladesh --Manuscript Draft.docx [file pgph.0006732.s007.docx]

**Manuscript Draft**

Responses are satisfactory.
